# Protocol for profiling *in vitro* intratumor heterogeneity using spatially annotated single-cell sequencing

**DOI:** 10.1016/j.xpro.2023.102447

**Published:** 2023-07-14

**Authors:** Myrthe M. Smit, Kate J. Feller, Li You, Miao-Ping Chien

**Affiliations:** 1 Department of Molecular Genetics, Erasmus University Medical Center, 3000 CA Rotterdam, the Netherlands; 2 Erasmus MC Cancer Institute, 3000 CA Rotterdam, the Netherlands; 3 Oncode Institute, 3521 AL Utrecht, the Netherlands

**Keywords:** Bioinformatics, Cancer, RNAseq, Microscopy

## Abstract

Here, we present a protocol for spatially annotated single-cell sequencing, a technique for spatially profiling intratumor heterogeneity with deep single-cell RNA sequencing and single-cell resolution. By combining live-cell imaging and photopatterned illumination, we describe steps to identify regions of interest in an *in vitro* tumor model, label the selected cells with photoactivatable dyes, and isolate and subject them to scRNAseq. This protocol can be applied to a range of cell lines and could be expanded to tissue sections.

For complete details on the use and execution of this protocol, please refer to Smit et al. (2022).^1^

## Highlights

• Spatially annotated scRNAseq can spatially profile intratumor heterogeneity
• Select cells of interest using live-cell imaging and profile them using deep scRNAseq
• Label and isolate cells of interest using photoactivatable dyes and cell sorting
• Spatially profile up to three regions of interest in tumor models and tissue sections

## Abstract (editor highlights)

Publisher’s note: Undertaking any experimental protocol requires adherence to local institutional guidelines for laboratory safety and ethics.

## Graphical abstract

**Figure:**
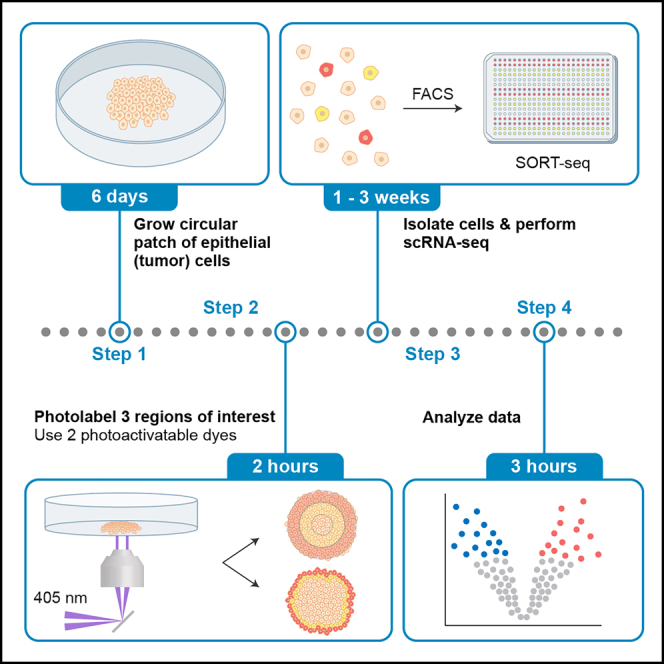

## Before you begin

Tumors are complex, heterogeneous tissues containing subpopulations of cells with distinct genetic, transcriptomic and proteomic profiles.^2^^,^^3^^,^^4^ Intratumor heterogeneity complicates effective cancer treatment, as rare tumor subpopulations can drive tumor progression, metastasis, and therapy resistance.^5^^,^^6^^,^^7^ Single cell sequencing is key to understanding the extent and implications of intratumor heterogeneity.^2^ However, since most protocols start from dissociated tissues, all information about the functional properties and spatial organization of cells is lost. In contrast, spatial transcriptomics methods capture the spatial heterogeneity in solid tissues but lack single cell resolution or have relatively low transcript counts, reducing their sensitivity to low abundance transcripts.^8^^,^^9^^,^^10^^,^^11^ Moreover, these methods often use fixed arrays of barcoded primers to capture transcripts, yielding uniform sampling across the tissue. Defining regions of interest (ROIs) based on microscopically observable features could be highly advantageous, as these ROIs could be sampled in more detail and studied at higher (single cell) resolution.

We describe spatially annotated single cell sequencing, a method to spatially profile intratumor heterogeneity with deep scRNAseq and single cell resolution.^1^ Users can manually or automatically detect up to three ROIs using live-cell imaging, which are then labeled, isolated, and subjected to scRNAseq. We apply our technology to study intratumor heterogeneity in the *in vitro* tumor model described by McFaline-Figueroa et al*.*^12^ When non-transformed MCF10A epithelial cells are grown in a 2D circular patch, cells at the edge of the patch undergo a confluency-dependent epithelial-to-mesenchymal transition (EMT), an important driver of intratumor heterogeneity.^13^^,^^14^^,^^15^ This spatial heterogeneity resembles *in vivo* findings, as tumor cells in the leading edge of head and neck squamous cell carcinomas express partial-EMT signatures.^3^ We therefore used a 2D circular patch of epithelial cells to represent and spatially profile intratumor heterogeneity. However, our versatile approach could have various applications, from traditional wound-scratch assays to profiling an *in vitro* tumor model. In principle, it should also be possible to spatially profile organoids and tumor sections, although we stress that our approach has currently only been validated in 2D cell cultures.

This protocol builds on the recently developed functional single cell sequencing (FUNseq) technology, which directly links tumorigenic phenotypes to causative phenotypes.^16^ FUNseq uses live-cell imaging and automated image analysis to identify tumor cells with a phenotype of interest. Patterned illumination with near-UV light labels these cells with a photoactivatable dye (i.e., “phototagging”), after which the labeled cells are isolated using flow cytometry and subjected to deep scRNAseq. Here, we multiplexed two photoactivatable dyes to annotate three confined ROIs. We incubate cells with one photoactivatable dye and illuminate the first ROI, after which cells are washed and incubated with a second photoactivatable dye. Another illumination cycle labels cells in the second ROI. Since both dyes were present in the cytoplasm during the second labeling cycle, we can distinguish three populations: unlabeled cells, cells labeled with the first dye, and cells labeled with both dyes. This approach might be extended to label more ROIs in a single sample with additional phototagging dyes, as long as the dyes are sufficiently spectrally distinct for efficient FACS sorting.

This protocol^1^ uses a custom-built microscope and scRNAseq technologies, but it can be adapted for resources used in different laboratories. For example, we recently used a similar approach to study the DNA-damage response using functional single cell proteomics, showcasing the versatility of FUNseq and related methods.^17^ In summary, spatially annotated single cell sequencing is a versatile approach that enables deep transcriptomic characterization of confined tumor regions.

### Cell seeding and culturing

**Timing: 6.5 days**

This section describes how to prepare the imaging dish and how to grow epithelial cells in a circular patch. All quantities listed in this protocol are for one 10-cm dish, user should scale up accordingly to include the required controls for photolabeling and flow cytometry tests (see corresponding sections).

***Note:*** Growth conditions have been optimized for MCF10A cells, the ideal number of cells used for seeding the patch and the time required to grow a semi-confluent monolayer of cells vary for different cell lines.

1. Prepare 400 μL of 0.1 mg/μL fibronectin solution in Dulbecco’s phosphate-buffered saline (DPBS; pH 7.0).

***Alternatives:*** Researchers can also coat glass-bottom dishes using different materials (e.g., collagen, poly-L/D-lysine, Matrigel®, etc.) or directly use uncoated imaging dishes and start at step 5.

2. Pipette 400 μL of the fibronectin solution on the glass area of a 35-mm glass-bottom imaging dish. Ensure that the entire glass surface is covered, and that the solution is evenly distributed.
3. Incubate for 20–30 min at 37°C in a 5% CO_2_ incubator.
4. Aspirate the fibronectin solution and wash the dish twice with 2 mL DPBS.
5. Harvest a 10-cm dish of MCF10A cells in regular culture media.

***Alternatives:*** Cell harvesting can be done using the researcher’s preferred subculturing method. We use trypsinization to collect cells, but one could also use Accutase™ treatment or cell scraping.

6. Centrifuge at 1000 × *g* for 5 min, aspirate supernatant and resuspend the pellet in 2 mL phenol-red free culture media to obtain a single-cell suspension.

**CRITICAL:** From this point onwards, use phenol-red free culture media when handling and growing the cells to prevent auto-fluorescence during imaging.

7. Obtain a cell count and carefully pipette 10,000 MCF10A cells on the center (glass area) of the imaging dish to form a small droplet.

**CRITICAL:** To form a homogenous, circular patch of cells, it is vital that this droplet is not disrupted at any point of the cell seeding protocol (Figure 1; problem 1).

8. Gently increase the total volume of the droplet to 20 μL with phenol-red free media while ensuring the droplet stays intact.

**Figure 1:**
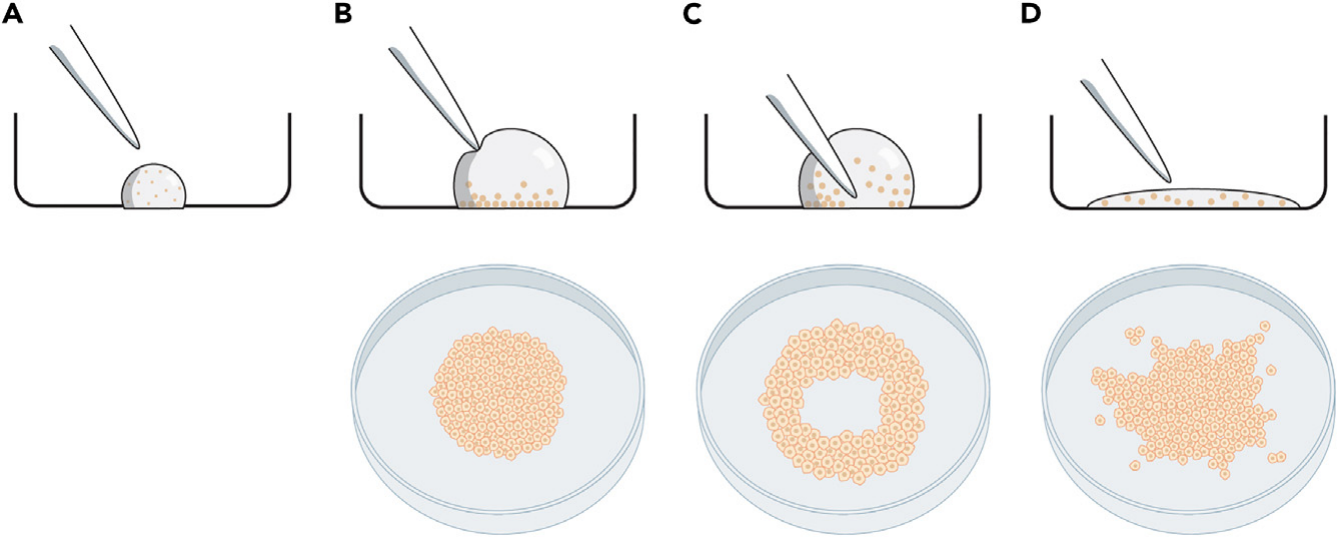
Illustration of successful and unsuccessful cell seeding approaches (A) First, a small droplet containing single-cell suspension is deposited on the glass area of the imaging dish. (B) Without disrupting this droplet, culture media is gradually added to prevent the droplet from drying up. Cells attach to the surface within the area of the droplet, forming a circular patch of cells. Bottom panel adapted from Smit et al.^1^ (C) If the culture media is added with too much force or if the pipette tip touches the surface of the dish, a hole will form in the center of the patch. (D) If the droplet is disrupted and the single-cell suspension spreads out over the surface of the dish, cells will form an irregularly shaped patch.
9. Incubate for 90 min at 37°C in a 5% CO_2_ incubator.

**CRITICAL:** Ensure that the droplet does not dry up during the incubation period. If necessary, carefully pipette extra media on top of the droplet.

**CRITICAL:** Be careful not to touch the surface of the dish when pipetting and handle the dish with care to ensure that the droplet stays intact.

10. Gently pipette 80 μL phenol-red free culture media on top of the droplet and incubate for 90 min at 37°C in a 5% CO_2_ incubator.
11. Gently pipette 100 μL phenol-red free culture media on top of the droplet and incubate for 2 h at 37°C in a 5% CO_2_ incubator. At this point, all cells should be attached to the fibronectin-coated surface.
12. Carefully aspirate culture media and wash the dish with 2 mL DPBS to remove any non-attached cells.
13. Add 2 mL phenol-red free culture media and incubate the cells for 6 days at 37°C in a 5% CO_2_ incubator. Refresh the growth media after 3 days.

***Note:*** The time required to grow the patch of cells varies per cell line. We aimed to obtain a patch of cells that covers approximately 25–50% of the glass area of the imaging dish (i.e., aim to grow a patch with a diameter of 10–15 mm). Please refer to Figure 1 and troubleshooting 1 to assess if a proper patch of cells was obtained.

### Preparing phototagging dye

**Timing: 15 min, to be performed on the day of photolabeling**

This section describes how to prepare the photoactivatable dyes used for the phototagging process.

***Note:*** The optimal dye concentration varies per cell line,^18^ it is recommended to test different concentrations before performing cell isolation and scRNAseq (troubleshooting 2).

14. Reconstitute photoactivatable Janelia Fluor 549 dye^18^ (“PA-JF549”) in DMSO to obtain a 2 mM stock solution. This solution can be aliquoted and stored at −20°C.

***Alternatives:*** Any photoactivatable dye can be used for the photolabeling process, as long as the dyes are sufficiently spectrally distinct to allow efficient flow cytometry sorting.

15. Reconstitute photoactivatable Janelia Fluor 646 dye^18^ (“PA-JF646”) in DMSO to obtain a 2 mM stock solution. This solution can be aliquoted and stored at −20°C.
16. Prepare 200 μL of 40 μM PA-JF549 solution in phenol-red free culture media.
17. Prepare 200 μL of 40 μM PA-JF646 solution in phenol-red free culture media.

**CRITICAL:** Phototagging dyes are light-sensitive, it is recommended to work fast and protect the (diluted) dyes from exposure to light.

## Key resources table

**Table:** 

REAGENT or RESOURCE	SOURCE	IDENTIFIER
**Chemicals, peptides, and recombinant proteins**		

Human plasma fibronectin purified protein	EMD Millipore	FC010
Dulbecco’s phosphate-buffered saline (DPBS), no calcium, no magnesium (pH 7.0)	Gibco	14190144
Hanks' balanced salt solution (HBSS), calcium, magnesium, no phenol-red (pH 6.7–7.8)	Gibco	14025092
DMEM/F12 culture media, no phenol red	Gibco	21041025
Donor equine serum	Gibco	16050-122
Penicillin/Streptomycin	Gibco	15140122
Epidermal growth factor	Thermo Fisher	AF-100-15-1MG
Hydrocortisone	Sigma/Merck	H-0888
Insulin	Sigma/Merck	I1882
Cholera toxin	Sigma/Merck	C8052
0.4% Trypan blue	Gibco	15250061
PA Janelia Fluor 549 Dye	Tocris	6149
PA Janelia Fluor 646 Dye	Tocris	6150
DMSO, anhydrous	Invitrogen	D12345
BD Draq7	BD Bioscience	BDB564904
0.5% Trypsin-EDTA, no phenol red	Gibco	15400054

**Critical commercial assays**		

SORT-seq	Single Cell Discoveries	N/A

**Deposited data**		

Raw scRNAseq data	Smit et al.^1^	GEO: GSE196245

**Experimental models: Cell lines**		

MCF10A-H2B-GFP	Reuven Agami Laboratory (Netherlands Cancer Institute)	N/A

**Software and algorithms**		

R	https://cran.r-project.org	V 4.1.1
Seurat	Hao et al.^19^	v 4.0.5; https://satijalab.org/seurat/articles/install.html
ClusterProfiler	Wu et al.^20^	V 4.0.5; https://bioconductor.org/packages/release/bioc/html/clusterProfiler.html
GSVA	Hänzelmann et al.^21^	V 1.40.1; https://www.bioconductor.org/packages/release/bioc/html/GSVA.html
CellphoneDB	Efremova et al.^22^	https://github.com/ventolab/CellphoneDB
CellComm	Lummertz da Rocha et al.^23^	https://github.com/edroaldo/fusca
Domino	Cherry et al.^24^	https://github.com/Elisseeff-Lab/domino

**Other**		

35 mm glass bottom dish with 20 mm micro-well	Cellvis	D35-20-1.5-N
Ultrawide Field-of-view Optical microscope	You et al.^16^	N/A

## Materials and equipment

### Ultrawide field-of-view Optical (UFO) microscope

• Details on the setup of the Ultrawide Field-of-view Optical microscope used for live-cell imaging and photolabeling are described in You et al.^16^
• ≥biological sample. We recommend to perform several phototagging tests with replicate samples before each experiment to achieve optimal photolabeling of the regions of interest. In general, ∼100 J/cm^2^ of 405 nm laser is sufficient to activate photoactivatable dyes.
• The code used to photopattern a circular patch of cells in three concentric layers is deposited online (see resource availability). Users can also script personalized code to label specific ROIs, as long as the sample contains at most three regions (for the current protocol) and each region contains enough cells to be sorted efficiently.

***Alternatives:*** While these experiments were performed on our custom-built UFO microscope, researchers can use any microscope suitable for live-cell imaging that is connected to a digital micromirror device (DMD) and a 405 nm laser to selectively illuminate regions of interest.

### MCF10A culture media

**Table:** 

Reagent	Final concentration
DMEM/F12 cell culture media, no phenol-red	N/A
Donor Equine Serum	5%
Penicillin/Streptomycin (10,000 U/mL)	1%
Epidermal growth factor	20 ng/mL
Hydrocortisone	500 ng/mL
Cholera toxin	100 ng/mL
Insulin (≥20 U/mg)	10 μg/mL
Total	**N/A**

***Note:*** store at 4°C for up to three months.

## Step-by-step method details

### Imaging and photolabeling

**Timing: 2 h**

This section describes how to photolabel three concentric rings in the circular patch of epithelial cells. The same protocol can be used to label the outer layers of this patch, or researchers can customize the photopatterning script to label any region of interest. ROIs can be selected based on bright-field or nuclear stain fluorescence images.

***Note:*** The photoactivatable dyes used here are cell permeable and retained in the cytoplasm for several hours. The rationale of the photolabeling approach is that we first incubate cells with one photoactivatable dye, after which the cells in the outer population are photoactivated and labeled with a single color. When we subsequently incubate cells with the second photoactivatable dye, the first dye is still present in the cytoplasm of the cells. Therefore, when the middle population is illuminated, both dyes are photoactivated and these cells are labeled with both dyes.

1. Aspirate culture media from the glass-bottom dish and wash cells with 250 μL phenol-red free culture media.
2. Pipette 200 μL 40 μM PA-JF646 solution on the glass area of the dish. Ensure that the patch of cells is fully immersed in the solution.
3. Incubate the cells for 20 min at 37°C.
4. Aspirate the PA-JF646 solution, wash cells with 250 μL phenol-red free culture media and add 250 μL phenol-red free culture media to the glass area of the dish.
5. Place the dish on the microscope stage and obtain a brightfield image of the patch of cells.

**CRITICAL:** It is essential to properly fix the position of the dish using stage clips, as any movement of the sample between the imaging and photolabeling steps will yield inaccurate results.

6. Using the photopatterning code, obtain a mask for the DMD to selectively illuminate the outer ring of the patch.
7. Load the mask on the DMD and phototag the outer ring with a 450 nm laser for 150 s (∼100 J/cm^2^).
8. Aspirate media and pipette 200 μL 40 μM PA-JF549 solution on the glass area of the dish. Ensure that the patch of cells is fully immersed in the solution.
9. Incubate the cells for 20 min at 37°C.
10. Aspirate the PA-JF549 solution, wash cells with 250 μL phenol-red free culture media and add 250 μL phenol-red free media to the glass area of the dish.
11. Place the dish on the microscope stage and obtain a brightfield image of the patch of cells.
12. Using the photopatterning code, obtain a mask for the DMD to selectively illuminate the middle ring of the patch.
13. Load the mask on the DMD and phototag the middle ring with a 450 nm laser for 150 s (∼100 J/cm^2^).
14. Obtain fluorescence images by exciting cells at approximately 549 nm and 646 nm (for PA-JF549 and PA-JF646, respectively) to assess the accuracy and efficiency of the photolabeling process (troubleshooting 2).

***Optional:*** To assess the effect of the photolabeling process on the viability of cells, prepare an extra glass-bottom dish of cells and illuminate the cells with a 405 nm laser with the same laser intensity and exposure time that is used in a specific experimental set-up. Continue with the optional steps from the cell isolation section.

**Pause point:** If one is labeling and isolating cells from multiple glass-bottom dishes, photolabeled dishes can be stored in a tissue culture incubator until all dishes are labeled and ready for sorting.

### Cell isolation

**Timing: 2 h**

This section describes the process to isolate photolabeled cells and prepare them for scRNAseq. FACS sorting is used to separate the patch of cells in three populations: unlabeled cells (the center of the patch), cells labeled with only PA-JF646 (the outer ring) and cells labeled with both PA-JF549 and PA-JF646 (the middle ring).

**CRITICAL:** Keeping cells in HBSS for prolonged periods of time affects cell viability. If one is sorting multiple dishes in a single run, we recommend limiting the time that cells are suspended in HBSS by trypsinizing the next dish only when one is almost done with sorting the previous dish.

15. Aspirate media and wash cells with 2 mL DPBS.
16. Pipette 250 μL Trypsin-EDTA without phenol red on the glass area of the dish (where cells were seeded).
17. Incubate until the cells are detached (∼20–30 min) at 37°C.

**CRITICAL:** From here on, use low retention pipette tips and tubes for handling the cells.

18. Dissociate the cells by carefully pipetting up and down and transfer cells to a 1.5 mL tube.
19. Rinse the glass area of the dish with 200 μL trypsin-EDTA without phenol red and transfer to the same tube.
20. Rinse the glass area of the dish with 200 μL HBSS with calcium and magnesium chloride (pH 7.0) and transfer to the same tube.
21. Gently pipette cell suspension up and down to further dissociate the cells.
22. Centrifuge cells for 4 min at 4000 × *g*.

***Note:*** The resulting pellet might be small and hard to see. Marking the outside of the tube before centrifuging helps to localize the pellet more easily.

**Figure 2:**
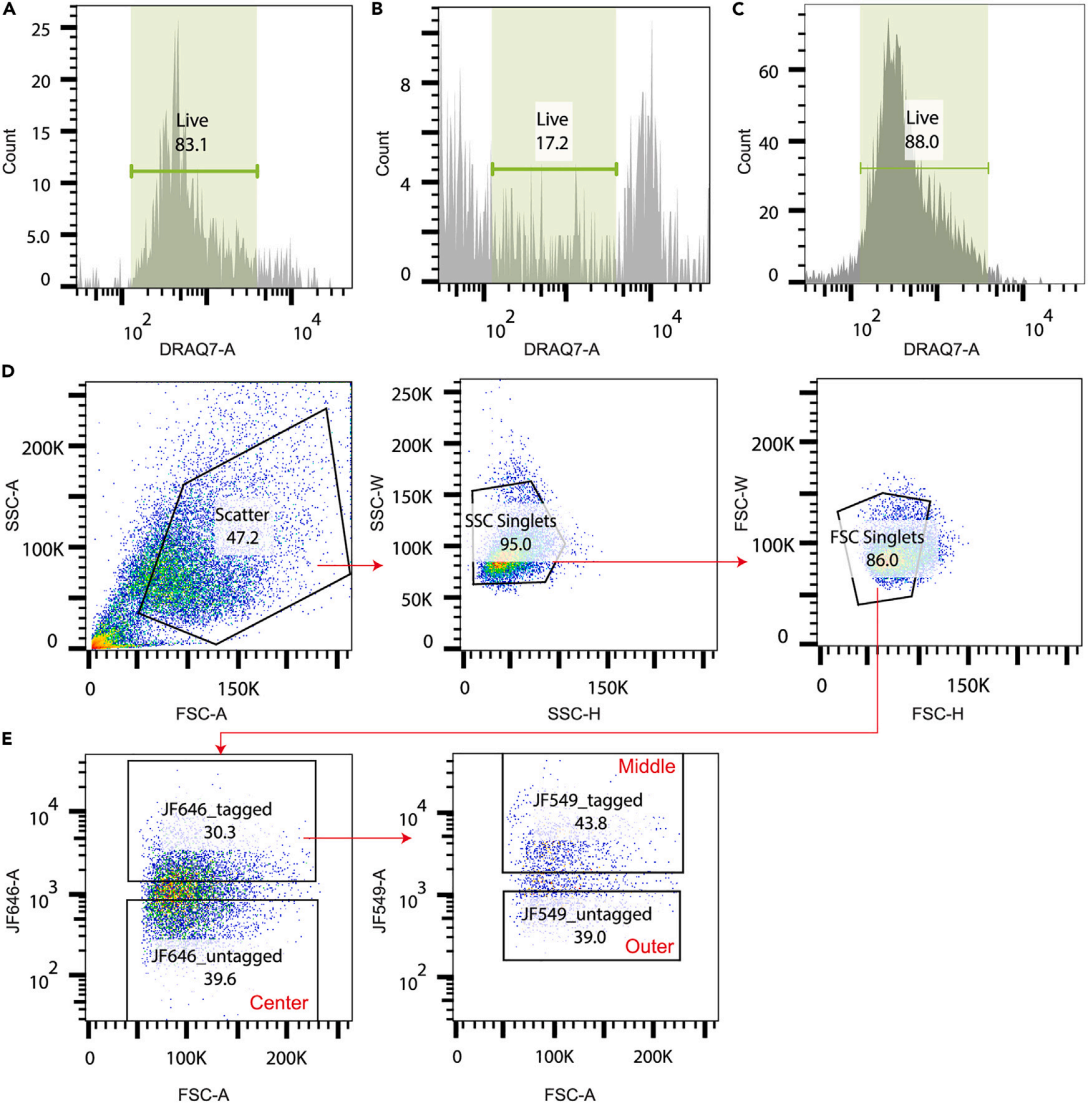
FACS gating strategy (A) Positive control (live cells) for DRAQ-7 live/dead staining. (B) Negative control (dead cells) for DRAQ-7 live/dead staining. (C) DRAQ-7 live/dead staining indicates that illuminating cells for 120 s with near-UV light does not alter cell viability. (D) Gating strategy to identify live, single cells. (E) Gating strategy to identify center, middle and outer populations (gated on live, single cells).

23. Aspirate supernatant and thoroughly resuspend cells in 400 μL HBSS to obtain a single cell suspension. Directly place on ice.
24. FACS sort the cells according to the machine’s user manual (Figure 2; troubleshooting 3).
  a. Sort live, single cells into the 384-well plates containing CEL-seq2 primers, RNA spike-ins and dinucleotide triphosphates (dNTPs) that are supplied by sequencing companies (i.e., Single Cell Discoveries in our study) or core facilities. Briefly centrifuge each plate before carefully removing the aluminum cover.
  b. Seal the plate with a new aluminum foil.
  c. Centrifuge for 1 min at 1000 × *g* to ensure cells are at the bottom of the wells and immediately place 384-well plates on dry ice.
  d. Plates can be stored for up to 3 months at −80°C until shipment to the sequencing company or facility.

**CRITICAL:** For each well, write down to which population the isolated cell belongs. This information is essential for the analysis of scRNAseq data. For most FACS machines, this sorting report can be downloaded after the sort is complete.

***Note:*** We outlined the FACS gating strategy in Figure 2. From the population of live, single cells, we identify cells that are negative for PA-J646, which originate from the center population. From the population of cells that are positive for PA-JF646, we identify cells that are positive for PA-JF549 (the middle population) or negative for PA-JF549 (the outer population).

***Note:*** The number of cells sorted into each population depends on the experimental setup. For our experiments, we sorted each dish of cells into one 384-well plate (i.e., approximately 120–150 cells per population). For statistical analysis, we recommend having approximately equal numbers of cells in the different populations.

***Optional:*** If assessing the effect of the photolabeling process on cell viability, first follow steps 15–22 and then continue with the steps outlined below:

25. Resuspend cells in 620 μL ice-cold HBSS buffer and add 3.1 μL 0.3 mM Draq7 viability dye.
26. Gently pipette to mix and incubate for 5 min at 37°C, while preventing exposure to light.
27. FACS sort according to the user manual, live cells should be negative for Draq7 fluorescence (Figure 2C).

### Single cell sequencing

**Timing: 1–3 weeks (depending on the facility used)**

This section describes how to profile the isolated cells using single cell RNA sequencing.

For library preparation and scRNAseq, our laboratory uses the SORT-seq platform^25^ offered by Single Cell Discoveries (Utrecht, The Netherlands). This sequencing approach is based on the Cel-seq2 technology.^26^ We sequenced our cDNA libraries at 150,000 reads/cell on the Illumina NextSeq 500 platform. In principle, researchers could use any plate-based scRNAseq technology that offers sufficiently high read depth.

## Expected outcomes

**Figure 3:**
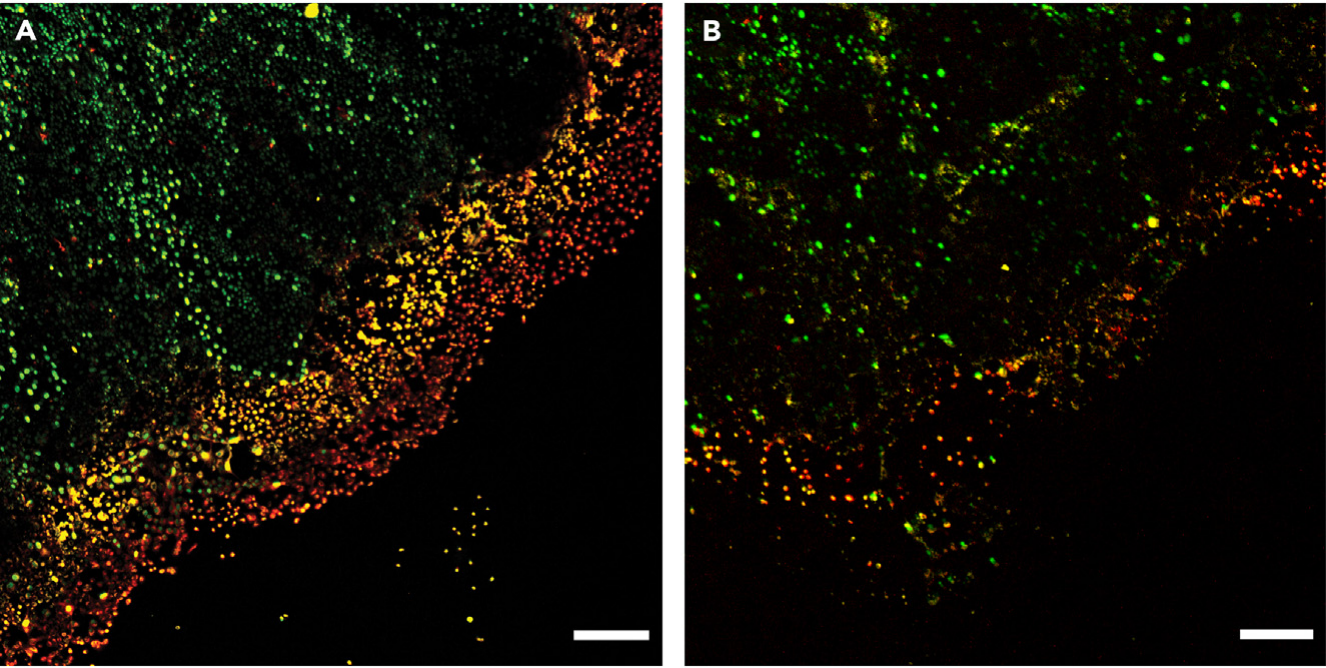
Example images for successful and unsuccessful photolabeling (A) The invading edge of the patch of cells is successfully labeled with PA-JF549 (yellow) and PA-JF646 (red). Labeled and unlabeled regions are clearly visible and well-separated. The outer edge is labeled only with PA-JF646, the middle region is labeled both with PA-JF549 and PA-JF646 and the center of the patch is unlabeled. Nuclei of the MCF10A cells are depicted in green. Figure reproduced from *Smit* et al.^1^ with permission. (B) Suboptimal photolabeling results in a “smear” of labeled cells, without clear separation of the different regions. In this example, the laser power was too low to properly activate the photoactivatable dyes. Scale bars represent 300 μm. For visualization purposes, we subtracted image background and adjusted image contrast in all channels of these composite images using ImageJ.

This protocol should efficiently photoactivate cells of interest, yielding a clear separation of photolabeled and unlabeled cells (Figure 3A). Expected cell yields after FACS vary per cell line and experimental setup. In our experience, it should be feasible to obtain at least 384 cells per glass-bottom dish of cells. If researchers handle the cells carefully and perform the isolation procedure quickly, we expect to have at least 70% healthy, single cells after scRNAseq (see below).

## Quantification and statistical analysis

This section describes the analysis of the scRNAseq data obtained using this protocol in Smit et al., 2022 Smit et al.^1^ This analysis is fully based on published R and Unix packages, for detailed instructions on their installation and usage readers are referred to the corresponding publications. Researchers can modify this analysis to suit their needs, sample data to perform the analysis is available at NCBI GEO DataSets (GSE196245).

1. Perform alignment and pre-processing of the scRNAseq^25^ data to obtain Poison-corrected Unique Molecular Identifier (UMI) counts.^25^
2. Generate a Seurat v4 object^19^ that contains the gene expression matrices.^19^ If necessary, combine different plates originating from a single patch of cells in one object.

**CRITICAL:** Ensure that cell names correspond to the 384-well number, so that one can retrieve to which population each cell belonged (based on the FACS data).

3. Add the population name as metadata for each cell.
4. Filter out ERCC spike-in genes (if used during scRNAseq).
5. Filter out cells containing low-quality scRNAseq data to ensure the downstream analysis is performed on live, single cells. To do this, select cells containing 2,000–9,000 features (genes) per cell and less than 40% mitochondrial genes. Researchers can expect to filter out 20%–30% of the cells using these criteria.

***Note:*** The exact thresholds for the quality control filtering depend on the cell line studied and the scRNAseq technology used. We recommend visualizing the distribution of the number of features per cell, the number of reads per cell and the percentage of mitochondrial genes to determine the optimal thresholds.

6. Normalize the UMI counts to obtain relative gene expression profiles for all cells. Our lab currently uses two different normalization functions: *SCTransform* is^27^ used for dimensionality reduction and the global-scaling NormalizeData function is used for all other downstream analysis.^27^
7. Perform cell-cycle scoring and regression using the *performPreprocessing* function with the set of G2/M and S phase markers supplied by Seurat.^4^ If necessary, perform batch correction of the gene expression data.

***Note:*** In our experience, SORT-seq results obtained from a single experiment do not require batch correction.

8. Perform a Principal Component Analysis (applied on the SCtransform assay) and determine how many principal components will be used for dimensionality reduction. In our analysis, we have used the first 40 principal components.
9. Perform a dimensionality reduction using the Uniform Manifold Approximation and Projection (UMAP) approach.^28^
10. Color UMAP visualization by population to assess the clustering of cells based on their spatial organization or by EMT score (see below) using Seurat’s *DimPlot* or *FeaturePlot* functions, respectively.
11. To calculate the EMT score for each cell, users can follow the approach from Sacchetti et al.^29^ by performing Gene Set Variation Analysis using the GSVA package^21^ (Table 1^30^).
  a. Calculate GSVA enrichment scores for epithelial and mesenchymal genes for each cell.
  b. Obtain a combined EMT score by subtracting the epithelial from the mesenchymal score.

**Table 1:** EMT marker genes from the nCounter PanCancer Progression Panel

Epithelial markers			Mesenchymal markers		
**AGR2**	ESRP1	PTK6	AKAP12	FBLN1	PDGFC
**AP1M2**	F11R	RAB25	AKAP2	FBN1	PLEKHO1
**ARHGAP32**	FAM174B	RBM47	AKT3	FERMT2	PLXNC1
**BCAS1**	FGFR3	S100A14	ANGPTL2	FGL2	PMP22
**CBLC**	FUT3	SCNN1A	ASPN	FHL1	PTGDS
**CD24**	GALNT7	SDC4	BGN	FLI1	PTGIS
**CD2AP**	GDF15	SH3YL1	BICC1	FN1	PTPRC
**CDH1**	GPR56	SLC44A4	BNC2	FSTL1	PTRF
**CDS1**	GRHL2	SORD	C1S	FXYD6	PTX3
**CEACAM1**	HDHD3	SPDEF	CALD1	GIMAP4	QKI
**CEACAM5**	IRF6	SPINT1	CAV1	GIMAP6	RUNX1T1
**CEACAM6**	KRT19	ST14	CCL8	GLYR1	SACS
**CKMT1A**	KRT7	TJP2	CD163	GREM1	SAMSN1
**CLDN7**	LAD1	TJP3	CDH11	GZMK	SERPINF1
**CXADR**	MUC1	TMEM30B	CDH2	HEG1	SERPING1
**CYB561**	MYO5C	TMPRSS2	CDK14	IGF1	SFRP1
**ELF3**	OCLN	TMPRSS4	CEP170	IL10RA	SLIT2
**EPCAM**	OVOL2	TOM1L1	CHRDL1	ISLR	SNAI2
**EPN3**	PLS1	TSPAN1	CLEC2B	ITM2A	SPARC
**EPS8L1**	PPL	VAMP8	CLIC4	JAM2	SPARCL1
**ERBB2**	PRR15L	VAV3	COL5A2	JAM3	SRGN
**ERBB3**	PRSS8		COL6A1	KCNJ8	SYNE1
			COL6A2	KIAA1462	TCF4
			CRISPLD2	LHFP	TNC
			CSF2RB	LOX	TNS1
			CTSK	LY96	TPM2
			CXCL12	MAF	TWIST1
			CXCL13	MEOX2	VCAM1
			CXCR4	MFAP4	VCAN
			CYP1B1	MMP2	VIM
			DCN	MPDZ	VSIG4
			DDR2	MRC1	WIPF1
			DPT	MS4A4A	WWTR1
			DPYSL3	MS4A6A	ZCCHC24
			ECM2	MYLK	ZEB1
			EMP3	NAP1L3	ZEB2
			ENPP2	NR3C1	ZFPM2
			EVI2A	OLFML2B	
			FAP	PCOLCE

***Alternatives:*** We used the EMT markers from the Nanostring nCounter PanCancer Progression Panel^30^ (Table 1) for the GSVA analysis, but researchers can also use custom sets of epithelial and mesenchymal markers.

***Note:*** Different approaches to calculating EMT scores are used in the field, for an overview of various methods please refer to Chakraborty et al*.*^31^

12. Identify differentially expressed genes between the inner and outer populations using Seurat’s *findMarkers* function applied on the RNA assay. Fold change (FC) and significance threshold can be set by the user, our analysis used a Wilcoxon rank-sum test to select genes with |log_2_(FC)| > 0.5 and a Bonferroni correct p-value < 1 × 10^−5^.
13. Perform an overrepresentation analysis using the ClusterProfiler v4 package^20^ to identify which gene sets are overrepresented in the different populations. Genes that are significantly upregulated in the outer and center populations can be compared to any gene set of interest (e.g., the MSigDB^32^ and and Wikipathways^33^ databases) using the *enricher* function with default parameters (one-sided Fisher’s exact test with Benjamini-Hochberg adjusted p-values).
14. To predict enriched ligand-receptor interactions between the photolabeled populations, users can perform a CellphoneDB analysis.^22^ This database contains information about receptor-ligand complexes and their interactions and can infer cell-cell communications from gene expression data. For more information and instructions, users are referred to https://www.cellphonedb.org.

***Alternatives:*** Users could also predict cell-cell interactions and downstream signaling networks using methods such as CellComm^23^ or Domino,^24^ which integrates scRNAseq data and information on protein-protein interaction networks to infer cell-cell communications and the signaling pathways that are activated as a result thereof.

## Limitations

While this protocol has been optimized for photolabeling a patch of MCF10A cells, the universality of the protocol remains to be tested. However, we have applied our original FUNseq protocol^16^ to a variety of cell types (including U2OS and HeLa cells, patient-derived head and neck squamous cell carcinoma cells and patient-derived glioblastoma cells), indicating that the process of photolabeling should be widely applicable.

Currently, the number of different regions that can be photolabeled in a single sample is limited to three. Researchers could try to increase this number by incubating cells with additional phototagging dyes, but one should first verify that these dyes are sufficiently spectrally distinct.

Another limitation of this protocol is its relatively low throughput in terms of the number of cells being analyzed. This means that the cells have to be sequenced at a higher read depth to ensure enough transcriptome coverage in the scRNAseq analysis. In theory, one might also sort the labeled and unlabeled cells into different tubes and subject these cells to droplet-based scRNAseq to achieve a higher throughput.

Finally, our UFO microscope has an ultrawide field-of-view (FOV), enabling us to image and photolabel the entire patch in a single FOV. In the case that a microscope with a smaller FOV is used for the experiments, users can image the patch in parts and stitch the resulting images together to identify the regions of interest.

## Troubleshooting

### Problem 1

Cells don’t grow in a homogeneous, circular patch; the patch has an irregular shape or contains holes (before you begin step 13; Figure 1).

### Potential solution

To ensure proper patch formation, users should pipette extremely carefully when seeding the cells on the imaging dish. The droplet of single cell suspension should remain intact and circular until all cells have attached to the surface (until before you begin step 13). If the surface tension is released (i.e., if the droplet is broken), cells will spread out on the dish and form an irregular shape. Additionally, if one pipettes the cell suspension or the additional media too vigorously, cells can be pushed away from the center of the droplet, leaving a hole in the patch.

### Problem 2

Cells of interest are not efficiently photolabeled, there is little to no increase in fluorescence compared to unlabeled cells (imaging and photolabeling step 14; Figure 3B).

### Potential solution

There may be several reasons for this problem and the optimal experimental setup for photolabeling varies per cell type. Conditions that we always optimize for different cell types include the phototagging dye concentration, the incubation time of the phototagging dye, the intensity and exposure time of the 405 nm laser. Additionally, if the cells in the center of the patch are overconfluent, the phototagging dye might not properly be taken up by these cells.

### Problem 3

After defining the gating scheme for FACS, there are little cells left for sorting or the cells in the sample have deteriorated (cell isolation step 24.a).

### Potential solution

Since the circular patch often contains only a limited number of cells, we have addressed this issue by seeding an extra imaging dish with a homogeneous monolayer of approximately 100,000 cells. Half of this extra dish was phototagged and trypsinized using the same conditions as the actual sample. We then used these cells to set the gating scheme on the FACS machine, after which we applied the same scheme to subsequent dishes. In this way, one can quickly update the gating scheme if necessary and use (almost) all cells in a sample for the actual sorting process.

## Resource availability

### Lead contact

Further information and requests for resources and reagents should be directed to and will be fulfilled by the lead contact, Miao-Ping Chien (m.p.chien@erasmusmc.nl).

### Materials availability

This study did not generate new unique reagents.

### Data and code availability

Original data have been deposited to NCBI GEO with accession number GSE196245. The code for photopatterning the patch of cells is available at https://sourceforge.net/projects/funseq/files/Sptial%20Transcriptomes/.

The code for bioinformatics analysis is available on GitHub (github.com/ChienMPLab/SpatiallyAnnotatedFUNseq)
